# Novel drug candidates against antibiotic-resistant microorganisms: A review

**DOI:** 10.22038/IJBMS.2023.71672.15593

**Published:** 2024

**Authors:** Jing-Sheng Lim, Yoke-Yen Chai, Wei-Xin Ser, Aniqah Van Haeren, Yan-Hong Lim, Tarshiiny Raja, Jhi-Biau Foo, Sharina Hamzah, Renukha Sellappans, Hui Yin Yow

**Affiliations:** 1 School of Pharmacy, Faculty of Health and Medical Sciences, Taylor’s University, Subang Jaya, Malaysia; 2 Medical Advancement for Better Quality of Life Impact Lab, Taylor’s University, 47500 Selangor, Malaysia; 3 Department of Pharmaceutical Life Sciences, Faculty of Pharmacy, Universiti Malaya, Kuala Lumpur, Malaysia

**Keywords:** Antibacterial agents, Antibacterial drug, resistance, Antibiotic resistance, Antimicrobial agent, Drug discovery, Microbial, Pharmacology

## Abstract

Antibiotic resistance is fast spreading globally, leading to treatment failures and adverse clinical outcomes. This review focuses on the resistance mechanisms of the top five threatening pathogens identified by the World Health Organization’s global priority pathogens list: carbapenem-resistant *Acinetobacter baumannii*, carbapenem-resistant *Pseudomonas aeruginosa*, carbapenem-resistant, extended-spectrum beta-lactamase (ESBL)-producing *Enterobacteriaceae*, vancomycin-resistant *Enterococcus faecium* and methicillin, vancomycin-resistant *Staphylococcus aureus*. Several novel drug candidates have shown promising results from *in vitro* and *in vivo* studies, as well as clinical trials. The novel drugs against carbapenem-resistant bacteria include LCB10-0200, apramycin, and eravacycline, while for *Enterobacteriaceae*, the drug candidates are LysSAP-26, DDS-04, SPR-206, nitroxoline, cefiderocol, and plazomicin. TNP-209, KBP-7072, and CRS3123 are agents against *E. faecium*, while Debio 1450, gepotidacin, delafloxacin, and dalbavancin are drugs against antibiotic-resistant *S. aureus*. In addition to these identified drug candidates, continued *in vitro* and *in vivo* studies are required to investigate small molecules with potential antibacterial effects screened by computational receptor docking. As drug discovery progresses, preclinical and clinical studies should also be extensively conducted on the currently available therapeutic agents to unravel their potential antibacterial effect and spectrum of activity, as well as safety and efficacy profiles.

## Introduction

Antibiotic resistance poses a grave threat to the future of healthcare and medicine. Although the emergence of antibiotic resistance is a natural phenomenon that occurs over time due to genetic mutations, the overuse and inappropriate use of antibiotics has accelerated the evolution of bacteria and driven us towards a post-antibiotic era whereby treatment failure has been reported in the management of bacterial infections. According to Teoh *et al.*, approximately 50% of prescribed antibiotics are deemed unnecessary, exposing bacteria to the mechanisms of drugs which has inevitably caused an increased need for new novel compounds to replace ineffective drugs (1). The development of novel antibiotics is further retarded by the low return on investment as the cost of developing an antibiotic is around US$1.5 billion in 2017, which only generates a return of $46 million per year (2). The intrinsic resistance of chromosomal mutation developed is defined as a trait that is shared universally within a bacterial species, is independent of previous antibiotic exposure, and is not related to horizontal gene transfer (3). These mutated resistant bacteria may also spread the resistance genes to previously susceptible bacteria. This is termed horizontal evolution, whereby the attainment of genetic material from resistant organisms to other susceptible bacterial species allows them to acquire the resistance mechanism learned. This spread of antimicrobial resistance (AMR) is rapid due to the presence of plasmids and other transferrable gene components such as integrons, transposons, and genome islands (4). 

The antibiotic resistance phenomenon spreads globally causing an increase in major treatment failures and unwanted clinical outcomes throughout the globe. Given this posing a vital threat to the health of the public worldwide, the World Health Organization (WHO) released a global priority pathogens list (Global PPL) in February 2017 that comprised three categories: critical, high, and medium priority pathogens (5). This initiative is aimed to provide a framework for global research and the development of new drugs to overcome these high-resistance developing bacteria. This list also highlights the mechanism of gram-negative bacteria that can transfer genetic material among other non-resistant bacteria in addition to their internal resistance. 

**Figure 1:**
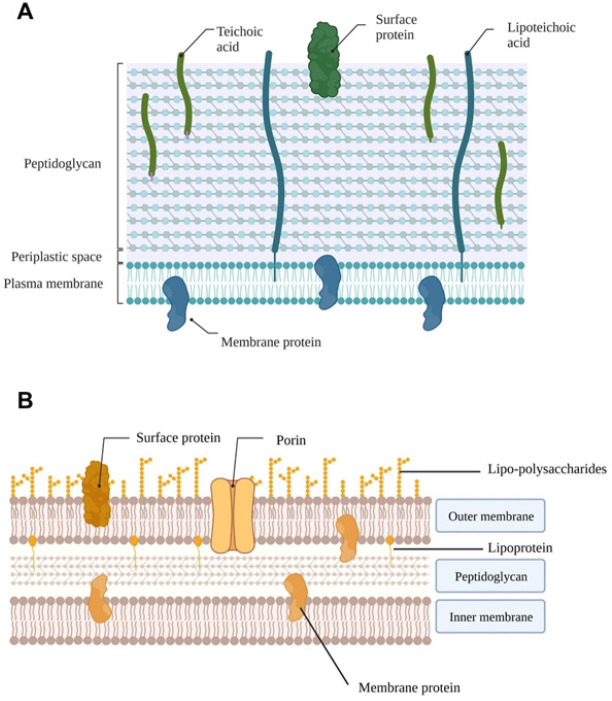
Schematic representation of bacterial cell wall

Gram-negative bacteria are found to be the most concerning due to their unique characteristic of having an outer membrane component that overlays the peptidoglycan layer compared to gram-positive bacteria which are missing an external membrane (Figure 1). This is a major reason attributed to intrinsic resistance developed by gram-negative bacteria. As an example, the use of vancomycin in gram-negative is deemed useless because the drug is unable to pass through the external membrane compared to β-lactams that travel through porins or hydrophobic drugs that diffuse across. Therefore, any changes that occur to the external membrane of gram-negative bacteria can inherently cause resistance to drugs, making gram-negative bacteria have superior resistance to antibiotics than gram-positive bacteria (6).

In this review, the resistance mechanisms of the top 5 threatening pathogens from the WHO global priority pathogens list were discussed, including carbapenem-resistant *Enterobacteriaceae* which are *Acinetobacter baumannii *(CRAB) and carbapenem-resistant* Pseudomonas aeruginosa *(CRPA*),* carbapenem-resistant, extended-spectrum beta-lactamase (ESBL)-producing* Enterobacteriaceae *(CRE), as well as vancomycin-resistant* Enterococcus faecium *(VRE) and methicillin, vancomycin-resistant* Staphylococcus aureus *(MRSA/VRSA)*. *These pathogens are also most known and referred to as the ESKAPE bacteria, an acronym used to refer to nosocomial pathogens encompassing both gram-negative and gram-positive species: *E. faecium,* *S. aureus*,* K. pneumoniae*,* A. baumannii*, *P. aeruginosa,* and *Enterobacter* species, which are able to “escape” the antimicrobial effects of clinically used antibiotics. Besides that, this review also highlighted promising novel compounds to be developed in order to overcome the resistance against the abovementioned resistant bacteria.

***Overview: Antibiotic resistance mechanisms***

**Figure 2:**
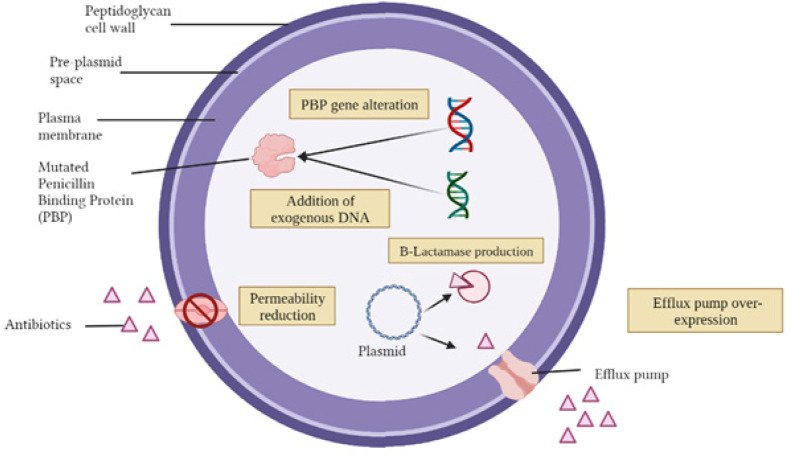
Schematic illustration of general bacterial antibiotic-resistant mechanisms

ESKAPE pathogens have possessed various antibiotic resistance mechanisms including drug inactivation or alteration, modification of drug binding sites, reduction of intracellular drug accumulation, and biofilm formation (8). Drug inactivation or alteration is extensively used by ESKAPE bacteria to develop resistance against antibiotics. This can be carried out by the production of enzymes that modify or deactivate the antibiotics. For example, β-lactamases, which can inactivate the β-lactam ring structure which is essential for antibiotics, such as penicillin, cephalosporins, monobactams, and carbapenems. These β-lactamases produced by gram-negative bacteria are listed according to two classifications: (i) Ambler Scheme (molecular classification) and (ii) Bush-Jacoby-Medeiros system (9). These systems aim to classify the enzymes based on their functions, which introduces the opportunity to classify the various enzymes according to their selective resistance to different β-lactam antibiotics. Although the Ambler Scheme molecular structure classification is easier and more common to use, a functional classification should be the preferred method heading into future research and development of antibiotics, which group these β-lactamase enzymes according to their specific hydrolytic and inhibition properties for better selectivity towards infections in the clinical setting (8). Other than gram-negative bacteria, gram-positive bacteria possess a similar resistance mechanism via the inactivation of antibiotics due to enzymatic hydrolysis by β-lactamases. Secondly, bacteria are resistant to antibiotics by reducing the susceptibility and affinity of the antibiotics toward the active site of the target protein, penicillin-binding protein (PBP), either by the addition of exogenous DNA or alteration of the PBP gene. Over-expression of efflux pump and reduction in membrane permeability can also further reduce antibiotic concentrations (10)(Figure 2).

***Acinetobacter baumannii*** **and** ***Pseudomonas aeruginosa***

*A. baumannii* is the most common *Acinetobacter* species associated with hospital-acquired infections worldwide, causing opportunistic infections of the skin, bloodstream, urinary tract, and other soft tissues in critically ill patients in the intensive care unit (ICU)(11). On the other hand, *P. aeruginosa* is an opportunistic pathogen that is a leading cause of morbidity and mortality in cystic fibrosis patients and immunocompromised individuals (12).

*Antibiotic resistance mechanism of A. baumannii*

Carbapenems such as doripenem, imipenem, and meropenem are reserved foremost agents for treating both gram-negative and positive bacterial infections*. *However, there is an increasing trend of carbapenem resistance in the hospitals of South and Southern Asia, especially *in A. baumannii*-calcoaceticus complex (AB) isolates (13). Three primary mechanisms can cause reduced carbapenem susceptibility in *A. baumannii*: production of carbapenemases, expression of multidrug efflux pumps, and reduced expression or mutations in porin channels and external membrane proteins (Table 1). The most critical mechanism of resistance involved the hydrolysis of drugs, which was caused by a combination of diverse intrinsic and acquired carbapenemases, a β-lactamase that hydrolyzes the activity of carbapenem antibiotics. Besides that, chromosomal oxacillinase (OXA-51 and its derivatives), usually expressed at low levels, also increases carbapenem resistance when up-regulated following the insertion of the element IS*Aba1* or IS*Aba9* (13). Carbapenem resistance is also reported in class A carbapenemases from *Serratia marcescens* enzymes (SME) and imipenem-hydrolyzing β-lactamase (IMI) families, known from *Serratia* and *Enterobacter* isolates but they usually remain susceptible to extended-spectrum cephalosporins (14). Class B metallo-β-lactamases (MBLS) have a wide, deadly, and strong range of carbapenem hydrolyzing activity and are resistant to all β-lactam drugs except for monobactams. These enzymes require a water molecule and a zinc divalent cation to inactivate the β-lactam structure. Class C β-lactamases whereby *A. baumannii *has intrinsic AmpC cephalosporinase and are not carbapenemases. But its overproduction together with an efflux system with or without decreased external membrane permeability can contribute to carbapenem resistance. On the other hand, class D or OXAs β-lactamases can hydrolyze extended-spectrum cephalosporins and carbapenems (10).

The expression of multidrug efflux pumps is another mechanism of antibiotic resistance. Compared with external membrane porins which facilitate antibiotic uptake, efflux systems primarily remove the amount of antimicrobials by pumping them out of the cell. There are four classes of efflux pumps, which are the resistance nodulation division (RND) superfamily, the major facilitator superfamily (MFS), the multidrug and toxic compound extrusion (MATE) family, and the small multidrug resistance (SMR) family transporters (15). AdeABC, AdeFGH, and AdeIJK are efflux pumps from the RND family that highly contribute to carbapenem resistance. Zhu *et al.* confirmed that relative expression levels of AdeB in carbapenem-resistant *A. baumannii* were 10.4 to 62.3 times higher than carbapenem-sensitive *A. baumannii *(16). These RND family pumps are typically structured to have a transporter protein on the internal membrane, a membrane fusion protein (MFP), and an OMP channel. Of all the genes, the specific gene of *AdeABC *produces the highest carbapenem resistance. 

On the other hand, alteration of bacterial envelope permeability due to the reduced expression or mutations in porin channels and external membrane proteins allows the transportation of antibiotics across the external membrane. Reduced expression of porins, such as 29-kDa protein (CarO or carbapenem-associated OMP), HMP-AB, and OmpW lead to carbapenem resistance in *A. baumannii* (17).

*Antibiotic resistance mechanisms of P. aeruginosa*

*P. aeruginosa* is found as intestinal normal flora responsible for ICU-acquired pneumonia infections in immunocompromised patients. It has developed multiple resistance mechanisms similar to *A. baumannii* with some exceptions as summarized in Table 1. Firstly, MBLs are also significant carbapenemases found in *P. aeruginosa, *such as mostly spread VIM, followed by IMP. Regional spreading of genes that encode for MBLs are identified such as *São Paulo MBL-1 (SPM-1), German imipenemase (GIM-1), Australian imipenemase (AIM), Central Alberta MBL (CAM), Dutch imipenemase (DIM), Florence imipenemase (FIM), Hamburg MBL (HMB), São Paulo MBL (SPM), SIM, *and *NDM*. Other β-lactamases of *P. aeruginosa *resistant to carbapenem apart from class B are Ambler class A enzymes KPC and GES/IBC. All mentioned enzymes are identical to the isolation from *A. baumannii*, except for OXA type class D carbapenemases that are rarely reported in *P. aeruginosa, *but there are emerging strains reported from Spain, India, the United Kingdom, and Belgium (18).

On the other hand, the most found efflux pumps observed in *P. aeruginosa *are the multidrug efflux system AB-Outer membrane protein M (MexAB-OPrM), which comprises the MexB pump, the MexA linker lipoprotein, and the OprM exit portal. MexAB acting synergistically with altered permeability of the external membrane increases the intrinsic resistance of *P. aeruginosa *towards multiple drugs*. *This leads to the next mechanism of resistance which is diminished external membrane permeability. OprD external membrane porin allows carbapenems to enter the bacteria, however, mutations of the OprD gene have led to porin loss and down-regulation. This loss of OprD porin is commonly observed to be significant and specific towards carbapenem resistance in *P. aeruginosa* (19).

*Novel drugs against antibiotic-resistant A. baumanii and P. aeruginosa*

*Apramycin*

Treatment with aminoglycosides is limited by its narrow therapeutic index and side effects. However, the structure of apramycin (EBL-1003), featuring a bicyclic sugar moiety and a mono-substituted deoxystreptamine that is well-defined from other aminoglycosides, contributes to its unique properties. Other than its ability to impair bacterial protein translocation, apramycin will not be inactivated by modifying enzymes, is highly selective to bacteria mitochondrial ribosomes, and has fewer side effects compared to clinically approved aminoglycosides. Given its advantageous characteristics, apramycin is considered a desirable choice in human therapeutics (20).

Kang *et al.* conducted a study by comparing aminoglycosides that have been approved for human therapeutic use with a variety of resistant strain sets. The MIC_50/90 _values of apramycin for *A. baumannii* and *P. aeruginosa* were 8/32 mg/l and 16/32 mg/l, respectively (20). Its MIC_50/90_ for *A. baumannii *were 8-fold lower than gentamicin, tobramycin, and amikacin. However, the MIC values of *P. aeruginosa *were not significantly different (20) (Table 2). In quality control strain, apramycin used for *P. aeruginosa* was consistently in range and had reliable MIC determinations within the range sets (20). A study of murine lung infection further investigated the effectiveness of apramycin with strain AR bank #0282 and reported > 99% or > 99.99% reduction of the CFU counts with 5 or >5 mg/kg of EBL-1003, respectively (21). Furthermore, a single dose of 125 mg/kg EBL-1003 in CRAB-infected mice resulted in an AUC of 339 h×μg/ml in plasma and 299 h×μg/ml in ELF, suggesting a favorable lung penetration of 88% and an even distribution pattern (21).

*Eravacycline*

**Figure 3:**
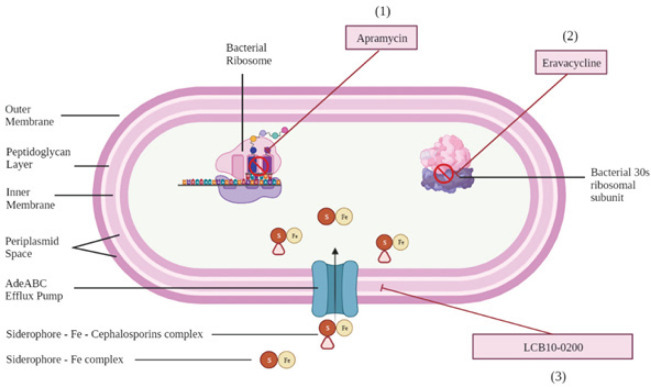
Mechanisms of action of novel drugs against antibiotic-resistant *Acinetobacter baumannii* and *Pseudomonas aeruginosa*

The United States Food and Drug Administration (FDA) has approved eravacycline used for treating complicated intra-abdominal infections in adults. Eravacycline, a novel synthetic fluorocycline antibiotic with the same structure as tigecycline, has been approved by the FDA for use against carbapenem non-susceptible *A. baumannii.* As a fluorocycline antibiotic, it inhibits bacterial protein synthesis by binding to the 30s ribosomal subunit (Figure 3)(22). 

An *in vitro* study was accomplished with around 286 *A. baumannii* isolates tested with anti-*Acinetobacter *reference drugs, such as beta-lactams, tetracyclines, fluoroquinolones, aminoglycosides, and colistin (22). The MIC_50/90_ of eravacycline was 0.5/1 mg/l, thus possessing a favorable bacteriostatic effect against the antibiotic-resistant strains. It has a lower MIC_50/90_ value compared to tigecycline, minocycline, and doxycycline with 1/2, 4/8, and 32/≥64 mg/l, respectively. Furthermore, eracacycline was found to have same MIC_50/90_ values in OXA-type bacteria, including OXA-23, OXA-58, OXA-40, and OXA-51 carbapenemase *A. baumannii*, with MIC_50/90_ 0.5/1, 0.5/0.5, 0.25/1 and range of 0.125 – 0.5 mg/l, respectively. In addition, the MICs for isolates with major international clonal lineages (IC strains) showed no significant difference compared to OXA-type isolates. The MIC_50/90_  for IC 1 strain and IC 2 strains was 0.5/0.5 and 0.5/1.0 mg/l, respectively; whereas the MICs value was 0.25/1 mg/l for the non-clustering strain. Overall, eracacycline with a MIC of ≤1 mg/l was shown to inhibit the growth of 96.5% of carbapenem-resistant *A. baumannii* isolates (22)(Table 2).

Furthermore, a Phase 3 clinical trial (NCT01844856) was conducted on 541 participants with complicated intra-abdominal infection (cIAI) to evaluate the efficacy and safety of eravacycline in comparison with ertapenem. The cure rate for the microbiological intent-to-treat population was 86.8% for the eravacycline treatment group and 87.6% for the ertapenem treatment group, with a difference of -0.80% (23). In addition, eravacycline also demonstrated higher cure rates compared to meropenem for cIAI in a randomized controlled study (NCT02784704) (Table 2) (24). The findings highlight that the treatment with eravacycline was non-inferior to meropenem in adult patients with cIAI, including infections caused by resistant pathogens.

*LCB10-0200 (GT-1)*

LCB10-0200 (also known as GT-1) is a new siderophore-cephalosporin drug developed to overcome the carbapenem-resistant *A. Baumanni and P. Aeruginosa*. This drug uses the ‘Trojan horse’ strategy by conjugating a siderophore with modified cephalosporin to increase the influx of antibiotics into bacterial cells (25). While LCB10-0200 is still undergoing clinical trials and has not been approved by the FDA, its potential to overcome antimicrobial resistance is summarized in Table 2 (26). It is proposed that the siderophores could increase their membrane permeability, which in turn increases the antimicrobial activity of the conjugated cephalosporin (25). Although LCB10-0200 was initially thought to primarily function as an antibiotic enhancer, it has been reported to possess noticeable antibacterial properties on its own. However, the exact mechanism of its antibacterial effect remains unclear, and further investigation is needed to elucidate its mechanism of action.

LCB10-0200 alone showed potent activity against meropenem-resistant *A. baumannii* and *P. aeruginosa* at MIC≤4 mg/l for 84.3% of isolates (ADC-, OXA-, and VIM-producing *A. baumannii and P. aeruginosa*). Although it was observed that a slightly higher percentage of isolates (86.3%) have their growth impaired with the MIC of ≤4 mg/l, it did not have any significant difference in effect on the tested isolates (25). It was more effective in countering KPC-, OXA-, non-fermenting gram-negative-type producing *P. Aeruginosa* and *A. Baumannii* compared to other drug molecules used. It had MIC_50/90 _values of 1/4 mg/l and 0.5/32 mg/l for KPC-producing *A. baumannii* isolates and *P. aeruginosa* isolates, respectively; while the MIC_50/90 _values for OXA-type and non-fermenting gram-negative type were 0.5/4 mg/l and 0.5/16 mg/l, respectively. There was no resistance observed during the *in vitro *study (25). 

The efficacy of the compound was also evaluated with a murine model against the systemic infections caused by *P. Aeruginosa*. The infected mice were administered 4 dose levels of LCB10-0200 or ceftazidime subcutaneously after the first and fourth hours of infection.  The MIC_(LCB10-0200/Ceftazidine)_ values for *P. aeruginosa* PAO 1, 1912E, R1023, and ARC3509 were 0.25/0.25 mg/l, 0.25/2 mg/l, 0.125/16 mg/l, and 0.5/64 mg/l, respectively. The outcome strongly indicates that LCB10-0200 was more effective and potent than ceftazidime which warrants the future investigation of the drug (27).

***Enterobacteriaceae***

*Enterobacteriaceae*, including species such as *Escherichia coli*, *Klebsiella spp.*, and *Enterobacter spp.*, are common multidrug-resistant bacteria that cause pneumonia, bloodstream infections, and urinary tract infections (UTIs) (10).

*Antibiotic resistance mechanisms of Enterobacteriaceae*

*Enterobacteriaceae* are known to produce ESBLs that have the ability to render wide-spectrum cephalosporins, monobactams, and penicillin inactive. These β-lactamases including class A temoniera (TEM)-1, TEM-2, and sulfhydryl reagent variable (SHV)-1, are among the most produced β-lactamases by *Enterobacteriaceae*. Gene mutations encoding for those enzymes have led to the emergence of new β-lactamases, with the capability to hydrolyze third-generation cephalosporins (10). In addition,* Enterobacteriaceae *can express other types of ESBLs such as CTX-Munich (CTX-M) which can inactivate cefotaxime, as well as AmpC β-lactamases which can inactivate third-generation cephalosporins. Hence, the use of clavulanic acid and other β-lactamase inhibitors is often ineffective against these lactamases as indicated in Table 1. Unlike *P. aeruginosa* and *A. baumannii*, OXAs are rarely expressed by *Enterobacteriaceae* (10).

Other than ESBL-producing, there are two classes of carbapenem-resistant *Enterobacteriaceae *(CRE), namely carbapenemase-producing CRE (CP-CRE) and non-carbapenemase-producing CRE (non-CP-CRE) (10). These carbapenem-hydrolyzing genes found in transferrable genetic components, such as integrons, transposons, plasmids, and insertion sequences, are the main reasons for carbapenem resistance, which permits horizontal sharing of genes to and among various bacterial species (28).

In CP-CRE, there are five major carbapenemases, including (1) *Klebsiella pneumonia carbapenemase *(KPC), class A serine based β-lactamases; (2) class B, New Delhi metallo-β-lactamases (NDM); (3) Verona integrin encoded metallo-β-lactamase (VIM); (4) class D, OXA or OXA-48-like carbapenemases; and (5) IMP, active on imipenem (10).

In non-CP-CRE, resistance toward carbapenems is identified as ESBL and/or AmpC β-lactamase producing. However, the production of ESBL and AmpC β-lactamases alone are insufficient to confer resistance, yet the resistance can be acquired via synergistic interaction with another mechanism, including (i) Absence of significant external membrane porin groups, such as OmpC and OmpF. These external membrane porins are also referred to as OmpK36 and OmpK35, respectively. This mechanism is frequently seen in CRE, as it decreases the external membrane permeability, leading to a decrease in drug uptake through the bacterial cell wall; (ii) Overproduction of efflux pumps such as AcrAB-TolC. It is a commonly found multidrug tripartite efflux pump comprising *acrA*, *acrB*, and *tolC* genes which encode for a membrane fusion protein of the periplasm, a transporter of the internal membrane, and a protein for the external membrane, respectively. It is a member of the RND superfamily and works synergistically with other mechanisms to increase resistance (28)(Table 1).

*Novel drugs against antibiotic-resistant Enterobacteriaceae*

*LysSAP-26*

**Figure 4:**
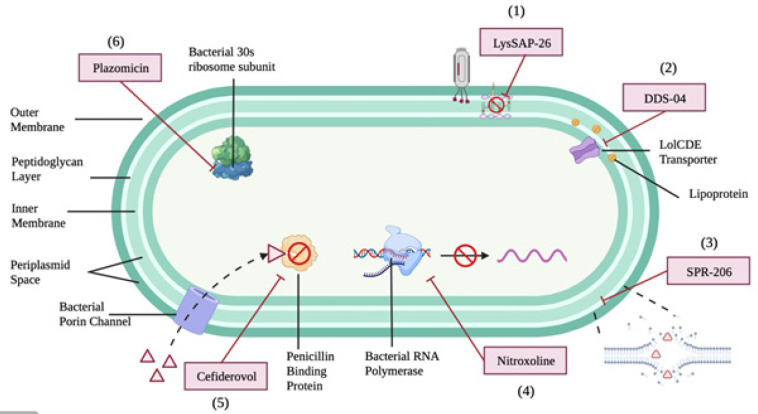
Mechanisms of action of novel drugs against antibiotic-resistant *Enterobacteriaceae*

LysSAP-26 is a promising *in vitro* medication against CRE, exerting antibacterial activity by creating pores in the cell wall via peptidoglycan digestion, leading to cell lysis caused by extensive high osmotic pressure (Figure 4). The gene for LysSAP-26 was extracted from the bacteriophage SAP-26 genome, and it has since been constructed and purified. LysSAP-26 had demonstrated a favorable *in vitro* bacteriostatic effect against *K. pneumoniae* strain (KCTC 2208) and *E. coli* strain (ATCC 25922) with a MIC of 20 µg/ml. The average observed MIC for both carbapenem-resistant* K. pneumoniae* (CRKP) and carbapenem-resistant and/or cephalosporin-resistant *E. coli* (CREC) was 20 µg/ml and 35 µg/ml respectively, thus, supporting the potential effect of LysSAP-26 against carbapenem-resistant *Enterobacteriaceae* (29). However, a LysSAP-26 possessed a modest bactericidal effect as the minimum bactericidal concentration for both CRKP and CERC was more than 80 µg/ml (29)(Table 3). 

In 2019, Summit Therapeutics Inc. suggested DDS-04, a novel drug composed of three sub-derivatives, as a bactericidal agent that inhibits LolCDE and impedes bacterial lipoprotein transport as illustrated in Figure 4. Since the drug target is clinically unexploited, the risk of pre-existing resistance and cross-sensitivity is low. An *in vitro* study for three different derivatives was performed on CRKP and CREC (30). The reported MIC for *K. pneumoniae* ranged from 0.5 to 4 µg/ml, with a MIC_90_ of 1 to 2 µg/ml. For *E. coli*, the MIC was 0.5 to 2 µg/ml, with a MIC_90_ of 0.5 to 1 µg/ml, indicating a favorable bacteriostatic effect. DDS-04 also showed a low frequency of resistance, with a range of 10^-9^ to 10^-10^ under the conditions of 4 to 16 times the MIC, which was an additional benefit of the compound for possessing a minimal risk of antibiotic resistance development. Moreover, DDS-04 was found to reduce bacterial burden in a murine model of UTI, with three days of three times daily IV 60 mg/kg leading to a significant reduction of *E. coli* UTI89 in urine from 10^7^ CFU/ml to 10^10^ CFU/ml (Table 3)(30).

*SPR-206*

SPR-206, a novel analog of polymyxin developed by Sper Therapeutics Inc. has been proposed as a direct-acting intravenous (IV) potentiator against CRE. An *in vitro* study involving a total of 101 KPC-, NDM-, and OXA-producing strains of *K. pneumoniae*, *E. coli*, *E. cloacae*, and *A. baumannii* showed that SPR-206 exhibited potent activity against most of the isolates (Table 3). For NDM-producing *Enterobacteriaceae*, the MIC_50_ and MIC_90_ were 0.125 µg/ml and 0.25 µg/ml, respectively, while for KPC-2-producing *Enterobacteriaceae*, the MIC_50_ and MIC_90_ were 0.125 µg/ml and 0.5 µg/ml, respectively (31). The antibacterial activity and toxicity of SPR-206 were further confirmed in another *in vitro* study, where the MIC values against *E. coli* IHMA558090, *E. coli* ATCC 25922, and *K. pneumoniae* ATCC 13882 were 8 µg/ml, 0.125 µg/ml, and 0.125 µg/ml, respectively, with lower cytotoxicity (32).

In an immunocompetent murine model with ascending UTI infection, SPR-206 showed promising clinical efficacy. The MIC of SPR-206 against *E. coli *ATCC 700928 and *E. coli* UTI89 was 0.03 µg/ml and 0.125 µg/ml, respectively, with a dosing of 4 mg/kg three times daily for three days leading to 3.05 CFU/g of *E. coli* ATCC 700928 and 3.11 CFU/g of *E. coli* UTI89 reductions of bacterial burden in mouse kidney (33). A Phase 1 randomized clinical trial to assess the safety and tolerability of SPR-206, was registered back in 2018 (NCT03792308), but no results have been published to date. Additionally, two Phase 1 studies (NCT04865393 and NCT04868292) are currently ongoing to investigate the intrapulmonary safety and pharmacokinetics profiles of SPR-206 (34, 35).

*Nitroxoline*

Other than the above-mentioned novel drugs, nitroxoline which was licensed back in 1954 for lower UTI was identified as a promising candidate to be studied. It was not included in treatment guidelines until 2016 due to a lack of data on resistance rates, MIC distributions, and the European Committee on Antimicrobial Susceptibility Testing (EUCAST)-confirmed break-points. Due to its low utilization in clinical practice, carbapenem-resistant *Enterobacteriaceae* was found to be susceptible to nitroxoline (36). Nitroxoline exerts a bacteriostatic effect by chelating the divalent cations required for bacterial RNA polymerase, thus impairing RNA biosynthesis. Based on an *in vitro* study carried out on 150 CRE isolates, nitroxoline possesses an encouraging killing effect with a MIC_50_ of 8 µg/ml and a MIC_90_ of 16 µg/ml (37). In another *in vitro* study with 146 ESBL producing *E.Coli, *the observed diameter of the inhibition zone was 11–30 mm and the MIC ranged from 2 to 64 µg/ml with MIC_50_ of 4 µg/ml and a MIC_90_ of 16 µg/ml (36)(Table 3).

*Cefiderocol (S-649266)*

Cefiderocol (S-649266) is a novel siderophore cephalosporin that was approved by the FDA in 2019. It demonstrated *in vitro* activity in suppressing carbapenem-resistant *Enterobacteriaceae* through its Torjan-Horse killing effect (entering bacteria via bacterial porin channel) with a MIC_90_ of 0.5-1 µg/ml (38). A Phase 2 clinical trial (NCT02321800) involving 452 hospitalized participants with complicated UTI was conducted in 2015-2016 to assess the efficacy and safety of intravenous cefiderocol. After receiving 2 g of cefiderocol by IV injection four times daily for 7 to 14 days, the microbiological eradication (ME) and clinical response obtained at Test of Cure (TOC) was 72.6% (39). The Phase 3 randomized clinical trial conducted in 2019 (NCT02714595) demonstrated that cefiderocol was effective against various infections with a 50.0% ME rate in hospital-acquired pneumonia (HAP), ventilator-associated pneumonia (VAP), healthcare-associated pneumonia (HCAP) and bloodstream infections (BSI)/sepsis BSI/sepsis, and a 52.9% ME rate in complicated UTI as summarized in Table 3 (40).

*Plazomicin*

A recently approved plazomicin that exerts bactericidal action against susceptible bacteria by binding to the bacterial 30S ribosomal subunit, has undergone three clinical trials to determine its clinical efficacy (41). In a Phase 2 clinical trial (NCT01096849) which compared the efficacy and safety between plazomicin and levofloxacin in treating complicated UTI and acute pyelonephritis, 85% of the ME population showed complete eradication at the TOC visit (Table 3). Whereas 80% of patients were clinically cured and only 6.5% experienced a relapse after 1 month (42). A Phase 3 study (NCT02486627) involving 609 patients reported that for the microbiologically modified intention-to-treat group, the clinical cure rate at day 5 was 88%, while at the TOC it was 81.7%. The clinical cure rate for ME groups on the other hand was 89.4% and 84.9% at day 5 and TOC, respectively. The rate of relapse was found to be as low as 1.8% (43). Similarly, another Phase 3 study (NCT01970371) reported that 85.7% of microbiological clearance was achieved at day 5 while the all-cause mortality rates at days 14 and 28 were 5.9% and 11.8%, respectively (44).

***Enterococcus faecium***

*E. faecium * is a gram-positive bacterium that has developed multi-resistance to antibiotic drugs such as vancomycin and the wide use of antibiotics has brought the evolution of *E. faecium *to a hospital-adapted pathogen (46).

*Antibiotic resistance mechanism of E. faecium*

As an antibiotic against the β-lactams resistant gram-positive bacteria, vancomycin has been used clinically in treating several severe infections, including meningitis, pneumonia, and sepsis (47). Since the emergence of vancomycin-resistant *Enterococcus *(VRE) in England and France in 1986, VRE has become a major nosocomial pathogen worldwide due to its colonization strategy, persistence in the environment, and genome plasticity (48). The most common risk factors of VRE are due to excessive use of broad-spectrum antibiotics, underlying disease, and admission to high-risk departments such as oncology, transplant, and ICU (49). There are several mechanisms of resistance involved, including the modification of the binding target and mutation of genes. The mechanism of resistance to vancomycin in *E. faecium* is potentially attributed to the modification of the binding target in vancomycin subsequently causing a change in the synthesis of peptidoglycan. The N-acetylmuramic acid (NAM) peptide terminal d-Ala-d-Ala is being replaced by d-Lac, and this eventually decreases the binding affinity between the precursors of the bacterial cell wall and the antibiotic (50)(Table 1). There are eight genotypes reported including *VanA, VanB, VanD, VanE, VanG, VanL, VanM*, and *VanN *with *VanA and VanB *being commonly found in hospital isolates. VRE management is challenging in the clinical setting as it depends solely on linezolid (49).

Other than vancomycin, linezolid resistance has also been demonstrated by *E. faecium*. According to the German National Reference Centre, it showed an increased development of resistance toward linezolid. The risk factor in developing linezolid resistance is attributed to prior exposure to linezolid (51). The most common mechanism of resistance of *E. faecium* toward vancomycin is associated with the mutation of G2576T and several variant genes in 23S ribosomal RNAs (52). In addition, mutations in ribosomal proteins such as *rplC* and *rplD* have been identified as mechanisms of linezolid resistance (53). More recently, it has been reported that genetic elements on a plasmid, including the *poxtA* gene, also contribute to this resistance (54). 

*Novel drugs against antibiotic-resistant F. faecium*

*TNP-2092*

**Figure 5:**
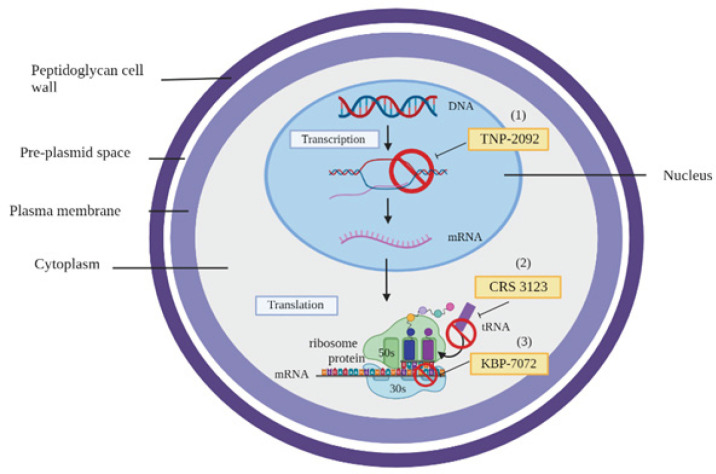
Mechanisms of action of novel drugs against antibiotic-resistant *Enterococcus faecium*

TNP-2092 is a rifampin-quinolone composite antibacterial composed of rifamycin SV and 4H-4-oxo-quinolizine pharmacophores that are covalently bonded (55). This novel drug inhibits RNA polymerase, DNA gyrase, and DNA topoisomerase IV, which are essential targets for bacteria residing in biofilms (Figure 5). In a study on mice with *C. difficile*-associated diarrhea (CDAD), TNP-2092 exhibited superior efficacy compared to metronidazole and vancomycin, with no recurrence detected following therapy at a minimum dose of 6.67 mg/kg (Table 4). Moreover, TNP-2092 demonstrated greater activity against specific gram-negative bacteria species than rifaximin, suggesting its possible effects against vancomycin-resistant *Enterococcus* (56).

A Phase 2 clinical trial (NCT03964493) was conducted on 118 participants with acute bacterial skin and skin structure infection (ABSSSI) suspected or confirmed to be caused by gram-positive pathogens. The primary outcome showed that the adverse event (AE) rate was lower in patients administered with TNP-2092 (46.2%) than in those given vancomycin (48.7%). Additionally, the early clinical response (ECR) of TNP-2092 was reported at 76.2%, which is higher than vancomycin (67.5%)(Table 4). Therefore, TNP-2092 exhibits higher efficacy, tolerability, and better safety profile in ABSSSI patients, making it superior to vancomycin (57).

*KBP-7072*

KBP-7072 is a novel tetracycline derivative with broad-spectrum antimicrobial properties against most pathogenic bacteria. It attaches to the main tetracycline recognition site on the 30S ribosomal subunit, implying that it functions as a protein synthesis inhibitor that inhibits A-site activation (Figure 5)(58). KBP-7072 is a novel third-generation tetracycline antibiotic that overcomes the widespread efflux and ribosomal protection resistance mechanisms that cause resistance in older-generation tetracyclines (59).

KBP-7072 demonstrated potent *in vitro* activity against many organisms, including *E. faecium.* The results revealed that KBP-7072 was active against 50 *E. faecium * strains (MIC_50/90_=0.03/0.03 mg/l; 100% inhibited at<0.12 mg/l), and its activity was not adversely affected by susceptibility or nonsusceptibility to vancomycin. Based on its MIC_50_ values, KBP-7072 was 4-fold more powerful than doxycycline (MIC_50/90_=0.12/8 mg/l) and 2-fold more active than minocycline (MIC_50/90_=0.06/16 mg/l), omadacycline (MIC_50/90_=0.06/0.12 mg/l) and tigecycline (MIC_50/90_=0.06/0.06 mg/l)(Table 4). Thus, the potent activity of KBP-7072 supports further clinical investigation in organisms infected with *E. faecium * in the future (59).

Three Phase 1 clinical trials (NCT02454361, NCT02654626, and NCT04532957) were conducted on healthy individuals to evaluate the safety, tolerability, and pharmacokinetics of a single dose of KBP-7072 but no results were posted upon completion of the study (Table 4) (59-61). Another clinical study was conducted to evaluate the dose-response and food-effect profile of KBP-7062 (62). The study involved 30 healthy individuals in fasting cohorts of 30 mg, 100 mg, 300 mg, 600 mg, and 1000 mg, as well as a group that received a fed dose of 100 mg (62). The results indicated that all treatment-emergent adverse events (TEAEs) were mild and were either unrelated or probably unrelated to the treatment (55). No serious adverse events were reported, and most of the adverse events were asymptomatic and resolved without intervention (62). Therefore, KBP-7072 exhibited a good safety profile and was well-tolerated at all doses. 

*CRS3123*

CRS3123 is a member of the 1-benzopyran class of organic compounds. This organic aromatic compound includes 1-benzopyran, a molecule composed of a benzene ring fused to a pyran with the oxygen atom in the 1-position (63). This new drug inhibits the synthesis of *C. difficile* toxin and spore formation by preventing *C. difficile* methionyl-tRNA synthetase (MetRS)(64). In an *in vitro* study, CRS3123 was effective against a wide range of *C. difficile* strains, such as BI/NAP1/027 strains (MIC range=0.5 to 1 μg/ml), and gram-positive cocci, such as *S. aureus*, *Streptococcus pyogenes*, *Enterococcus faecalis* and *E. faecium * (MIC_90_<1 μg/ml), but it was inactive against most of the gram-negative bacteria, such as *Lactobacillus* and *Bifidobacterium*. In short, CRS3123 was effective against *C. difficile* with high specificity and selectivity (65). The efficacy of CRS3123 was further evaluated in an *in vivo* *C. difficile* hamster gastrointestinal model (66). The result showed that CRS3123 inhibited *de novo* toxin production in high cell density (>108 cfu/ml) at a low concentration (1 mg/l), and it was superior to vancomycin (20 mg/l), while metronidazole had no effect under these conditions. Thus, CRS3123 is a potential agent against *C. difficile* infections as it inhibits toxin production and spore formation, therefore reducing the severity and spread of the disease (66).

The safety profile of CRS3123 was determined through Phase 1 clinical trial (NCT01551004) in healthy individuals by ascending administration of CRS3123 of 100 mg, 200 mg, 400 mg, 800 mg, and 1200 mg (Table 4)(67). The incidence of adverse events was 93.3% in the CRS3123 treated group and 90% in the placebo group. Nevertheless, no serious adverse events or immediate reactions were observed during the administration of CRS3123. The most reported adverse events were decreased hemoglobin, headache, and abnormal urine analysis. Overall, the mild to moderate adverse events observed in this study demonstrate that CRS3123 is well-tolerated within this dosage range. Therefore, these findings support further research and development of CRS3123 for the treatment of *C. difficile* infections (67, 68).

***Staphylococcus aureus***

*S. aureus *is one of the common human pathogenic microorganisms that can cause skin and soft tissue infections, endocarditis, osteomyelitis, bacteremia, and lethal pneumonia (70). Antibiotic resistance towards *S. aureus*, including methicillin-resistant *S. aureus* (MRSA), vancomycin-intermediate *S. aureus* (VISA), and vancomycin-resistant *S. aureus *(VRSA) have increased in incidence, resulting in morbidity and mortality.

*Antibiotic resistance mechanism of S. aureus*

*S. aureus *possesses methicillin resistance via the inactivation of methicillin by β-lactamase. This is due to the beta-lactam ring of the drug being hydrolyzed by the enzyme β-lactamase and leading to the destruction of the binding site of antibiotics (71). In addition, the alteration of PBP2 reduces the binding affinity of penicillin and increases the rate of release of the bound drug compared to the normal PBP2. However, the major cause of resistance of methicillin in *S. aureus* is through PBP2a; *mecA* encodes protein PBP2a, a unique transpeptidase that can take over the reaction of PBPs to form the cross-link in peptidoglycan to help bacterial cell wall formation (72, 73)(Table 1).

In addition to methicillin resistance, clinical cases have been reported on the emergence of vancomycin-resistant *S. aureus *(VRSA), which is completely resistant to vancomycin at a minimum inhibitory concentration (MIC) of ≥16 μg/ml. *S. aureus* strains with reduced susceptibility to vancomycin, with a MIC between 4-8 μg/ml, are referred to as vancomycin-intermediate *Staphylococcus aureus* (VISA) (74). Another type of vancomycin resistance, known as hetero-VISA, acts as the precursor to VISA and has varying susceptibilities to vancomycin, with MIC values ranging from <4 mg/l to ≥32 mg/l (75). The resistance mechanism in VISA involved the increase of cell wall turnover rate, resulting in an increase of non-cross-linked d-alanyl-d-alanine (D-ala-D-ala) side chains and reduced chances of vancomycin binding to intracellular target molecules (76). Furthermore, gene mutations such as *VanA* resistance can also lead to vancomycin resistance by substituting D-ala-D-ala with D-alanyl-D-Lactate, thereby reducing the affinity of vancomycin toward its binding site (77)(Table 1).

Various mechanisms of resistance are also reported for daptomycin. It involves the metabolism and dynamics of plasma membranes. This causes the change of components of the phospholipid, involving the phosphatidylglycerol (PG) lysyl-peptidoglycan and cardiolipin. When there is a decrease in the production of PG, this subsequently increases the conversion to lysyl-PG (L-PG), thus promoting bacterial resistance to daptomycin (78). In addition, many bacteria develop resistance to defensin-like cationic antimicrobial peptides (CAMPs) through the multiple peptide resistance factor *(MprF)*.* MprF* is a large membrane protein that reduces bacterial affinity to CAMPs through the modification of anionic phospholipid PG with l-lysine (79). Mutation of *MprF* shows an increase in L-PG which leads to an increase in the transportation of positively charged L-PG from inside to outside of the plasma membrane (Table 1). This eventually decreases the negative charge outside the cell. When the cell membrane surface becomes more positive, it reduces the chance of daptomycin’s positive charge binding to it (77). 

*Novel drugs against antibiotic-resistant S. aureus*

*Afabicin/Debio 1450*

**Figure 6:**
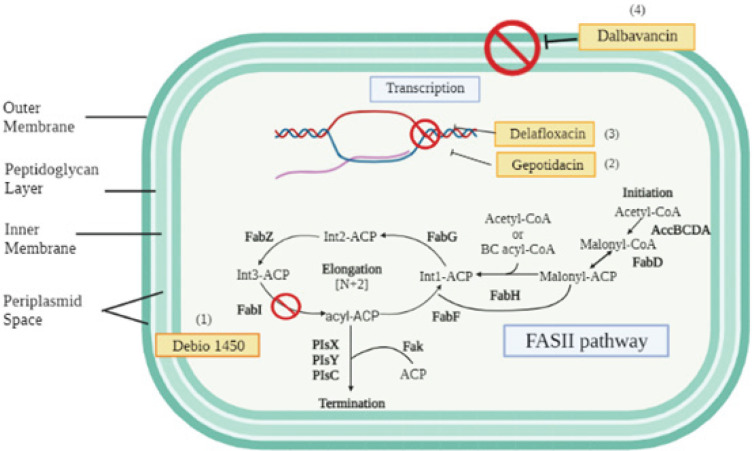
Mechanisms of action of novel drugs against antibiotic resistant *Staphylococcus aureus*

Afabicin, formerly known as Debio 1450 or AFN-1720, is a prodrug of afabicin desphosphono. It is the first drug of the novel antibiotic class that inhibits the synthesis of fatty acids (FASII) pathway in *staphylococci* bacteria by targeting enoyl-acyl carrier protein (FabI) reductase (Figure 6). FabI reduces enoyl-ACP to acyl-ACP in the final step of fatty acid chain elongation, which is important for the growth and survival of bacterial cells, and it is highly conserved across all staphylococcal species. By inhibiting FabI, ‘fabiotics’ represents a novel antibacterial class that has the potential to address the challenges of bacterial resistance.  

The MIC_90_ of Debio 1452 was 0.008 µg/ml against the MRSA isolates, which were collected in 2015 and 2016 (80) (Table 5). At the concentration of 0.06 µg/ml, it inhibited 99.4% of organisms. There was no cross-resistance of Debio 1452 with other antibacterial classes used in the treatment of infection caused by gram-positive pathogens (80). In an *in vivo* study, Debio 1452 was reported with significant effectiveness and high bone-to-plasma ratios of its active moiety in animal models infected with *S. aureus*-induced osteomyelitis (81).

Besides that, Debio 1450 also showed its efficacy in eradicating intracellular *S. aureus* in osteoblasts in patients who undergo hip replacement surgery. The findings from the clinical study (NCT02726438) showed that Debio 1450 penetrates well into bone tissue with a mean ratio of 2.88 when accounting for plasma and synovial fluid (82). Apart from this, a Phase 2 clinical trial (NCT02426918) involving 330 participants with acute bacterial skin and skin structure infections (ABSSSI) caused by* S. aureus *or MRSA was conducted to assess the efficacy of oral and intravenous Debio 1450 in comparison with oral linezolid and intravenous vancomycin. The early clinical response rate (ECRR) showed that Debio 1450 was non-inferior to vancomycin/linezolid with the outcome of 94.6% and 90.1% vs 91.1% (83)(Table 4).

Another Phase 2 randomized study (NCT03723551) was conducted in 2018 to assess the safety, tolerability, and efficacy of Debio 1450 in the treatment of participants with bone or joint infection caused by MRSA, methicillin-susceptible *Staphylococcus aureus* (MSSA) and coagulase-negative staphylococci (CoNS) and to compare it to standard of care. This study is ongoing and no results have been reported (84).

*Gepotidacin*

Gepotidacin is a novel drug that causes the inhibition of DNA gyrase and topoisomerase II through a unique mechanism that is different from the current approved therapeutic agent (Figure 6). Gepotidacin demonstrated activity against MRSA with MIC_50_ of 0.25 μg/ml and MIC_90 _of 0.5 μg/ml and its MIC values were not affected by other antibiotics such as linezolid, daptomycin, macrolide, clindamycin (inducible and constitutive), ceftaroline, and vancomycin resistance (85).

A Phase 3 clinical trial (NCT04020341) was conducted in 2019 to evaluate the therapeutic response of oral gepotidacin compared to oral nitrofurantoin for uncomplicated UTI in adolescent and adult female subjects (86). Parallelly, another Phase 3 clinical trial (NCT04010539) was conducted in 2019 to assess the efficacy and safety between oral gepotidacin and intramuscular ceftriaxone in combination with oral azithromycin in the treatment of patients with uncomplicated urogenital infection caused by *N. gonorrhoeae *(Table 5). However, these two studies are still in the process of recruiting participants (87).

*Delafloxacin*

In June 2017, FDA approved delafloxacin, a fluoroquinolone antibacterial drug that inhibits the activity of bacterial DNA topoisomerase IV and DNA gyrase for the treatment of ABSSSI caused by *S. aureus*, including MSSA and MRSA (Figure 6). Delafloxacin demonstrated superior activity against MRSA blood isolates (MRSABIs), VISA, VRSA, and daptomycin-non-susceptible strains (DNSSA) with the MIC_90_ and susceptibility (%) of 1mg/l and 68%, 1 mg/l and 40%, 4 mg/l and 7%, and 1 mg/l and 38%, respectively. (88). Besides that, the efficacy and safety of delafloxacin were also studied in Phase 3 clinical trials (NCT01811732) in patients with ABSSSI to compare with vancomycin/aztreonam. In the intention-to-treat (ITT) population, the objective responses at 48 to 72 hr for delafloxacin and vancomycin/aztreonam were 78.2% and 80.9%, respectively, with a mean difference of -2.6%. Investigator assessment at the follow-up visit was similar between the two groups (52.0% vs 50.5%) and late follow-up (70.4% vs 66.6%). Bacterial eradication of MRSA was 100% and 98.5% and adverse events were similar for both groups (3.70% and 3.68%) (Table 5)(89).

*Dalbavancin*

Dalbavancin is a lipoglycopeptide antibiotic that binds to the d-alanyl-d-alanine terminus of the stem pentapeptide in the bacterial cell wall peptidoglycan to prevent cross-linking and ultimately interferes with the synthesis of the bacterial cell wall (Figure 6). It was approved by the FDA for the treatment of ABSSSI, including MRSA in 2014.

A Phase 4 randomized clinical trial (NCT03426761) was conducted on 50 participants to evaluate the efficacy and safety of dalbavancin in patients with osteomyelitis or joint infections caused by gram-positive bacteria. As dalbavancin has a prolonged half-life, it may reduce the total cost and morbidity rate of native joint and prosthetic joint infections with an infusion every 14 days to complete the treatment with an approximation of 4 years. No results were posted as the study is ongoing (90).

Apart from this, a Phase 2b clinical trial (NCT04775953) was conducted in 2021 to compare dalbavancin to standard-of-care antibiotic therapy in patients with complicated bacteremia or right-sided native valve infective endocarditis (IE) caused by *S. aureus *who have cleared their baseline bacteremia. No results were posted as the study is still ongoing(91).

(A) Gram-positive bacterial cell wall. (B) Gram-negative bacterial cell wall. Created with BioRender.com. Adapted from (7) with permission

PBP gene alteration and addition of exogenous DNA altered the antibiotic binding affinity towards penicillin-binding protein (PBP). β-lactamase production contributed to drug modification or inactivation. Permeability reduction and over-expression of efflux pump reduced antibiotic concentration. Created with BioRender.com

**Table 1:** Summary of antibiotic resistance mechanisms for respective bacteria

**Drugs**	**Status**	**Mechanism of action**	**Study design**	**Outcome**	**Reference**
Apramycin	Under investigation	Inhibits bacterial ribosome and impairs bacterial protein translocation	*In vitro*	Favorable bacteriostatic effect against *A. baumannii *with MIC_50_ of 8 mg/L and MIC_90_ of 32 mg/L Favorable bacteriostatic effect against *P. aeruginosa *with MIC_50_ of 16 mg/L and MIC_90_ of 32 mg/L Higher potency as compared to other aminoglycosides with an 8-fold lower MIC value	(20)
			*In vivo* murine model infected with *A. baumannii *strain AR Bank #0282)	Reduced > 99.99% of CFU with a dose of > 5 mg/kg at q6h. Favorable lung penetration of 88%	(21)
**Fluorocycline - eravacycline**	FDA approved	Inhibits bacterial protein synthesis through binding to 30s ribosomal subunit.	*In vitro*	Favorable bacteriostatic effect against *A. baumannii *as MIC of ≤ 1 mg/L could inhibit the growth of 96.5% of carbapenem-resistant *A. baumannii* isolates	(22)
			Clinical trial (NCT01844856): A Phase 3 study to evaluate the efficacy and safety of eravacycline in comparison with ertapenem in patients with complicated intra-abdominal infections (n=541)	No non-inferiority was observed in eravacycline as compared to ertapenem in terms of cure rate as eravacycline has a cure rate of 86.8% and ertapenem has a cure rate of 87.6%.	(23)
			Clinical trial (NCT02784704): A Phase 3 study to evaluate the efficacy and safety of eravacycline in comparison with meropenem in patients with complicated intra-abdominal infections. (n=500)	No non-inferiority was observed in eravacycline as compared to meropenem inpatient as the primary endpoint clinical cure rate was 90.8% vs 91.2%; secondary endpoint clinical cure rate was 92.4% vs 91.6%; clinical cure rate = 87.5% vs 84.6%	(24)
**LCB10-0200**	Under investigation	Increases the antibiotics’ influx into bacteria by using the ‘Trojan Horse’ strategy	*In vitro*	Potent bacteriostatic effect against meropenem-resistant *A. baumannii* and *P. aeruginosa* as 84.3% of isolates stop growing under MIC ≤ 4 mg/L	(25)
			*in vitro* (KPC-, OXA-type, non-fermenting gram-negative type producing* P. Aeruginosa* and *A. Baumannii*)	Favorable bacteriostatic effect against KPC-producing *A. baumannii* isolates and *P. aeruginosa* isolates with MIC_50/90 _values of 1/4 mg/L and 0.5/32 mg/L Favorable bacteriostatic effect against OXA-producing strains with MIC_50/90 _values of 0.5/4 mg/L Favorable bacteriostatic effect against non-fermenting strains with MIC_50/90 _values of 0.5/16 mg/L	(25)
			*In vivo *murine model infected with *P. aeruginosa* PAO 1, 1912E, R1023, and ARC3509	LCB10-0200 demonstrated a more potent bacteriostatic effect as compared to ceftazidime with MIC values for *P. aeruginosa* PAO 1, 1912E, R1023, and ARC3509 of 0.25/0.25 mg/L, 0.25/2 mg/L, 0.125/16 mg/L and 0.5/64 mg/L, respectively.	(27)

OMP: outer membrane protein; ESBLs: extended-spectrum beta-lactamases; PBP: penicillin-binding proteins; L-PG: lysinylated-phosphatidylglyderol

**Table 2:** Novel drugs against antibiotic resistant *Acinetobacter baumannii* and *Pseudomonas aeruginosa*

**Drugs**	**Status**	**Mechanism of action**	**Study design**	**Outcome**	**Reference**
**LysSAP-26**	Under investigation	Phages and causes peptidoglycan digestion through lysis.	*in vitro*	Favorable bacteriostatic effect with 20 µg/ml of MIC, 20 µg/ml of average MIC for CRKP, and 35 µg/ml of average MIC for CREC isolates Modest bactericidal effect with > 80 µg/ml of MBC.	(29)
**DDS-04**	Under investigation	Inhibits LolCDE transporter and impairs intracellular lipoprotein transportation	*In vitro*	Favorable bacteriostatic effect against CRKP with MIC = 0.5 – 4 µg/ml and MIC_90_ = 1-2 µg/ml Favorable bacteriostatic effect against CREC with MIC of 0.5–2 µg/ml and MIC_90_ of 0.5–1 µg/ml Low risk of antibiotic resistance development with frequency of bacteria resistance of 10^-9^–10^-10^ under 4 to 16 folds of MIC	(30)
			*In vivo *murine model infected with *E. coli* UTI89	Favorable urine bacterial burden reduction from 10^7^ CFU/ml to 10^10^ CFU/ml after three days of three times daily IV dosing of 60 mg/kg	(30)
**SPR-206**	Under investigation	Impairs cell wall integrity via direct-acting IV potentiator.	*In vitro*	Potent bacteriostatic effect against NDM-producing *Enterobacteriaceae* with MIC_50_ of 0.125 µg/ml and MIC_90_ of 0.25 µg/ml Potent bacteriostatic effect against KPC-2-producing *Enterobacteriaceae *with MIC_50_ of 0.125 µg/ml and MIC_90_ of 0.5 µg/ml	(31)
			*in vitro*	Favorable bacteriostatic effect against *E. coli* IHMA558090 with MIC of 8 µg/ml Potent bacteriostatic effect against *E. coli* ATCC 25922 and *K. pneumoniae* ATCC 13882 with MIC of 0.125 µg/ml	(32)
			*In vivo *immunocompetent murine model infected with *E. coli *ATCC 700928 and *E. coli* UTI89	Favorable mouse kidney bacterial burden reduction. 3.05 CFU/g reduction for *E. coli *ATCC 700928 and 3.11 CFU/g reduction for *E. coli* UTI89	(33)
			Clinical trial (NCT03792308): A Phase 1 study to assess the safety, tolerability, and pharmacokinetics of different doses of SPR206 in healthy volunteers (n=94)	Study completed but results not available.	(45)
			Clinical trial (NCT04865393): A Phase 1, open-label study to assess the safety and pharmacokinetics profile of SPR206 in patients with varied renal function (n=40)	Study is ongoing (recruiting)	(34)
			Clinical trial (NCT04868292): A Phase 1, single-center, open-label study to determine intrapulmonary pharmacokinetics of SPR206 (n=30 healthy volunteers)	Study is ongoing (recruiting)	(35)
**Nitroxoline**	FDA approved	Inhibits bacterial RNA polymerase by chelating divalent cation	*In vitro*	Favorable bacteriostatic effect against carbapenem-resistant *Enterobacteriaceae *with MIC_50_ of 8 µg/ml and MIC_90_ of 16 µg/ml	(37)
			*In vitro*	Favorable bacteriostatic effect against ESBL-producing *E. coli *with MIC_50_ of 4 ug/ml and MIC_90_ of 16 ug/ml. The diameter of the inhibition zone was 11–30 mm	(36)
**Cefiderocol**	FDA approved	Enters cell via Trojan horse effect, binds and inhibits penicillin-binding proteins	Clinical trial (NCT02321800): A multicenter, double-blind, randomized, Phase 2 study to assess the efficacy and safety of IV S-649266 in comparison with imipenem/cilastatin for complicated UTI (n=452)	Favorable ME and clinical response at TOC of 72.6%.	(39)
			Clinical trial (NCT02714595): A multicenter, randomized, open-label Phase 3 study of S-649266 (n=152)	Favorable clinical cure at TOC for HAP/VAP/HCAP or BSI/Sepsis of 50.0% Favorable ME at TOC for complicated UTI of 52.9%	(40)
**Plazomicin**	FDA approved	Binds to the bacterial 30s ribosomal subunit	Clinical trial (NCT0109684): A Phase 2 study of the efficacy and safety of plazomicin compared with levofloxacin in treating complicated UTI (n=145)	Favorable eradication rate in the ME population of 85% and clinical cure rate of 80% Low relapse rate after 1 month of 6.5%	(42)
			Clinical trial (NCT02486627): A randomized, multicenter, double-blind Phase 3 study comparing the efficacy and safety of plazomicin with meropenem in the treatment of complicated UTI (n=609)	Favorable clinical cure of 88% for mMITT at day 5 and 81.7% at TOC Favorable clinical cure of 89.4% for ME at day 5 and 84.9% at TOC Low relapse rate after 1 month of 1.8%	(43)
			Clinical trial (NCT01970371): A Phase 3, multicenter, randomized, open-label study to evaluate the efficacy and safety of plazomicin compared with colistin in CRE-infected patients (n= 69)	Favorable CRE bacteremia clearance at day 5 of 85.7% Low rate of ACM was 5.9% at day 14 and 11.8% at day 28.	(44)

MIC: minimum inhibitory concentration; CRKP: carbapenem-resistant *K. pneumoniae*; CREC: carbapenem-resistant and/or cephalosporin-resistant *E. coli*; MBC: minimum bactericidal concentration; MIC_90_: minimum inhibitory concentration required to inhibit the growth of 90% of organisms; CFU/ml: colony-forming unit per milliliter; IV: intravenous; NDM: New Delhi metallo-beta-lactamase; MIC_50_: minimum inhibitory concentration required to inhibit the growth of 50% of organisms; KPC-2: Klebsiella pneumoniae carbapenemase-2; ME: microbiological eradication; TOC: test of cure; UTIs: urinary tract infections; HAP: hospital-acquired pneumonia; VAP: ventilator-associated pneumonia; HCAP: healthcare-associated pneumonia; BSI: bloodstream infections; mMITT: modified intention to treat group; CRE: carbapenem-resistant *Enterobacteriaceae*; ACM: all-cause mortality

(1) Apramycin inhibits bacterial ribosomes and impairs protein translocation. (2) Eravacycline inhibits bacterial 30s ribosome subunit and impairs bacterial protein biosynthesis. (3) LCB10-0200 increases the antibiotics’ influx into bacteria by using the ‘Trojan Horse’ strategy. Created with BioRender.com

(1) LysSAP-26 induces peptidoglycan digestion, resulting in loss of cell wall integrity and cell lysis. (2) DDS-04 inhibits LolCDE transporter and impairs intracellular lipoprotein transportation. (3) SPR-206 impairs cell wall integrity via direct-acting IV potentiator effect. (4) Nitroxoline inhibits bacterial RNA polymerase by chelating divalent cation. (5) Cefiderovol enters cells via the “Trojan Horse” effect and binds and inhibits penicillin-binding proteins. (6) Plazomicin binds to the bacterial 30s ribosomal subunit. Created with BioRender.com

**Table 3:** Novel drugs against antibiotic-resistant *Enterobacteriaceae*

**Drugs**	**Status**	**Mechanism of action**	**Study design**	**Outcome**	**Reference**
**TNP-2092**	FDA approved	Inhibits RNA polymerase, DNA gyrase, and DNA topoisomerase IV.	*In vivo* (mice infected with *C. difficile-associated* diarrhea)	No recurrence detected following therapy with minimum therapeutic dose of 6.67 mg/kg.	(56)
			Clinical trial (NCT03964493): A Phase 2 study to assess safety, tolerability, pharmacokinetic characteristics, and efficacy of TNP-2092 in ABSSSI adults suspected or confirmed to be caused by gram-positive pathogens. (n = 118)	ECR of TNP-2092 = 76.2%, ECR of vancomycin = 67.5% AE of TNP-2092 = 46.2% AE of vancomycin = 48.7% TNP-2092 is superior to vancomycin with better safety profile, tolerability, and efficacy.	(57)
**KBP-7072**	FDA approved	Binds to 30s ribosomal subunit and inhibits A-site activation.	*In vitro* (50 *E. faecium* strains)	KBP-7072 (MIC_50/90_ = 0.03/0.03 mg/L) Doxycycline (MIC_50/90_ = 0.12/8 mg/L) Minocycline (MIC_50/90_ = 0.06/16 mg/L) Omadacycline (MIC_50/90_ = 0.06/0.12 mg/L) Tigecycline (MIC_50/90_ 0.06/0.06 mg/L) KBP-7072 was more potent against *E. faecium* compared with doxycycline, minocycline, omadacyclin, and tigecycline with low MIC_50/90_	(59)
			Clinical trial (NCT02454361): A Phase 1 study to assess the safety, tolerability, and pharmacokinetics of a single dose of KBP-7072 in healthy people. (n = 46)	NA*	(60)
			Clinical trial (NCT02654626): A Phase 1 study to study multiple ascending doses of KBP-7072 in healthy people. (n = 16)	NA*	(69)
			Clinical trial (NCT04532957): A Phase 1 study to study multiple ascending doses to investigate the safety of KBP-7072 in healthy people. (n = 24)	NA*	(61)
**CRS3123**	Under investigation	Inhibit *C. difficile *methionyl-tRNA synthetase.	*In vitro* *(C. difficile* strains such as BI/NAPI/027)	CRS3123 = MIC 0.5 to 1 μg/ml *S. aureus, Streptococcus pyogenes, Enterococcus faecalis*, and *F. faecium* = MIC90s < 1 μg/ml	(65)
			*In vivo* *(C. difficile *hamster model)	CRS3123 = MIC 1 mg/L Vancomycin = MIC 20 mg/L Metronidazole = no effect CRS3123 was effective against *C. difficile *infection and was superior against vancomycin and metronidazole	(66)
			Clinical trial (NCT01551004): A Phase 1 study to determine the safety and pharmacokinetics of a single dose of CRS3123 in healthy adult volunteers. (n = 40)	AE in CRS3123 = 93.3% AE in placebo = 90% CRS3123 is well tolerated with mild to moderate adverse events	(67)

MIC: minimum inhibitory concentration; CRKP: carbapenem-resistant *K. pneumoniae*; CREC: carbapenem-resistant and/or cephalosporin-resistant *E. coli*; MBC: minimum bactericidal concentration; MIC_90_: minimum inhibitory concentration required to inhibit the growth of 90% of organisms; CFU/ml: colony-forming unit per milliliter; IV: intravenous; NDM: New Delhi metallo-beta-lactamase; MIC_50_: minimum inhibitory concentration required to inhibit the growth of 50% of organisms; KPC-2: *Klebsiella pneumoniae *carbapenemase-2; ME: microbiological eradication; TOC: test of cure; UTIs: urinary tract infections; HAP: hospital-acquired pneumonia; VAP: ventilator-associated pneumonia; HCAP: healthcare-associated pneumonia; BSI: bloodstream infections; mMITT: modified intention to treat group; CRE: carbapenem-resistant *Enterobacteriaceae*; ACM: all-cause mortality

(1) TNP-2092 inhibits RNA polymerase, DNA gyrase, and DNA topoisomerase IV in a novel mechanism. (2) CRS3123 inhibits methionyl-tRNA synthetase (MetRS). (2) KBP-7072 binds to 30S ribosomal subunit and inhibits A-site activation. Created with. BioRender.com

**Table 4:** Novel drugs against antibiotic resistant *Enterococcus faecium*

**Antibiotics**	**Bacteria**	**Mechanism of resistance**	**References**
**Carbapenem**	*A. baumannii*	Synthesis of class A, B and D carbapenemases Overproduction of efflux pumps (AdeABC, AdeFGH and AdeIJK) Mutation of OMPs and porins (29-kDa protein *HMP-AB* and *OmpW*)	(7) (12) (13)
	*P. aeruginosa*	Classes A, B, and D carbapenemases Overexpression of efflux pump (MexAB-OprM) Loss of outer membrane protein (OprD)	(14) (15)
	*Entero-* *bacteriacae*	Carbapenemases, ESBLs, and AmpC β-lactamases Overexpression of main efflux pumps (AcrAB-TolC)	(28) (11)
**Vancomycin**	*E. * *f* *aecium*	Alteration of vancomycin-binding target Mutation of genes (*vanA, vanB, vanD, vanE, vanG, vanL, vanM, and vanN*)	(49)
**Linezolid**		Mutation of G2576T (23 ribosomal RNAs) Mutation of ribosomal proteins (*rplC* and *rplD*) Plasmid encoded genetic elements such as the *poxtA* gene.	(54)
**Methicillin**	*S. aureus*	Inactivation by β-lactamases *MecA* encodes protein PBP2a	(73)
**Vancomycin**		Modification of vancomycin-binding target Thickened cell wall of peptidoglycan	(77)
**Daptomycin**		Increase L-PG production Mutation of *MprF*	

*Ongoing study (recruiting)

FDA: United States Food and Drug Administration; ABSSSI: acute bacterial skin and skin structure infection; ECR: early clinical response; AE: adverse event; MIC: minimum inhibitory concentration; MIC_50_: minimum inhibitory concentration by 50%; MIC_90_: minimum inhibitory concentration by 90%; NA:Not available; tRNA: transfer ribonucleic acid

**Table 5:** Novel drugs against antibiotic resistant *Staphylococcus aureus*

**Drugs**	**Status**	**Mechanism of action**	**Study design**	**Outcome**	**Reference**
**Debio 1450**	Under investigation	Inhibit the synthesis of fatty acids (FASII) pathway in staphylococci bacteria by targeting FabI, which is an enoyl-acyl carrier protein (ACP) reductase.	*In vitro* (MRSA)	MIC90 = 0.008 µg/ml	(80)
			Clinical trial (NCT02726438): A Phase 1 study to assess the effectiveness of oral Debio 1450 in patients who underwent hip replacement surgery. (n = 17)	Well penetrate bone tissue with a mean ratio of plasma: synovial fluid = 2.88	(82)
			Clinical trial (NCT02426918): A Phase 2 study to assess the efficacy of Debio 1450 orally and intravenously in comparison with oral linezolid and intravenous vancomycin in ABSSSI patients caused by *S. aureus* or MRSA. (n = 330)	ECRR for Debio 1450 80 mg/120 mg BID = 94.6% ECRR for Debio 1450 160 mg/240 mg BID = 90.1% ECRR for vancomycin/ linezolid BID = 91.1%	(83)
			Clinical trial (NCT03723551): A Phase 2 study to assess the safety, tolerability, and efficacy of Debio 1450 in the treatment of participants with bone or joint infection due to *S. aureus *and to compare it to the standard of care (n = 96)	NA*	(84)
**Gepotidacin**	Under investigation	Inhibits DNA gyrase and topoisomerase II by a unique mechanism.	*In vitro*	MIC_50_ = 0.25 ug/ml MIC_90_ = 0.5 μg/ml	(85)
			Clinical trial (NCT04020341): A Phase 3 study to evaluate the therapeutic response of oral gepotidacin compared to oral nitrofurantoin for uncomplicated UTI in adolescent and adult female subjects (n = 2055)	NA*	(86)
			Clinical trial (NCT04010539): A Phase 3 study to evaluate the efficacy and safety of gepotidacin compared with ceftriaxone plus azithromycin in the treatment of uncomplicated urogenital gonorrhea caused by *N. gonorrhea* (n= 600)	NA*	(87)
**Delafloxacin**	FDA approved	Inhibits the activity of DNA gyrase topoisomerase II and bacterial DNA topoisomerase IV	*In vitro*	Susceptibility to MRSABIs, VISA, VRSA, DNSSA= 68%, 40%, 7%, and 38%. MIC_90_ = 1 mg/L (MRSABIs, VISA, DNSSA) MIC_90_ = 4 mg/L (VRSA)	(88)
			Clinical trial (NCT01811732): A Phase 3 study to evaluate the efficacy of delafloxacin patients with ABSSSI to compare with vancomycin/aztreonam. (n = 660)	Delafloxacin vs vancomycin/aztreonam Objective response = 78.2% vs 80.9% Investigator assessment = 52.0% vs 50.5% Late follow-up = 70.4% vs 66.6% Bacterial eradication of MRSA = 100% vs 98.5% AE= 3.70% vs 3.68%	(89)
**Dalbavancin**	FDA approved	Binds to the d-alanyl-d-alanine terminus of the stem pentapeptide in the bacterial cell wall peptidoglycan.	Clinical trial (NCT03426761): A Phase 4 study to evaluate the efficacy and safety of dalbavancin in patients with osteomyelitis or joint infections caused by gram-positive bacteria (n = 50)	NA*	(90)
			Clinical trial (NCT04775953): A Phase 2 study to compare dalbavancin to standard-of-care antibiotic therapy in patients with complicated bacteremia or right-sided native valve infective endocarditis caused by* S. aureus* who have cleared their baseline bacteremia (n = 200)	NA*	(91)

*Ongoing study (recruiting)

FDA: United States Food and Drug Administration; NA: not available; MRSA: methicillin-resistant *Staphylococcus aureus*; MIC_50_: minimum inhibitory concentration by 50%; MIC_90_: minimum inhibitory concentration by 90%; ECRR: early clinical response rate; BID: two times daily; UTI: urinary tract infection; ABSSSI: acute bacterial skin and skin structure infections; VISA: Vancomycin Intermediate *Staphylococcus aureus*; VRSA: Vancomycin Resistance *Staphylococcus aureus*; MRSABI: methicillin-resistant *Staphylococcus aureus* blood isolates; DNSSA: daptomycin-non-susceptible strains; AE: adverse events

(1) Debio 1450 inhibits the synthesis of fatty acids (FASII) pathway by targeting FabI and inhibit the fatty acid chain elongation which is important for the growth and survival of bacterial cells. (2) Gepotidacin inhibits DNA gyrase and topoisomerase II. (3) Delafloxacin inhibits the activity of bacterial DNA topoisomerase IV and DNA gyrase. (4) Dalbavancin binds to the D-alanyl-d-alanine terminus of the stem pentapeptide in the bacterial wall peptidoglycan to prevent cross-linking and synthesis of bacterial cell wall. Created with BioRender.com

## Conclusion

In the pursuit of finding new ways to overcome antimicrobial resistance microbes, the discovery of new drug candidates is a strategy that has gained significant importance. Although the AMR strains could be tackled with multi-drug regimens or reserved drugs, the identification of novel molecules is an essential priority. Other than those short-listed drug candidates*, in vitro* and *in vivo* studies should also be conducted on small molecules in which their affinity towards drug targets could be screened by computational receptor docking. As drug discovery progresses, preclinical studies and clinical trials should also be extensively conducted on the currently available therapeutic agents to enhance the understanding of their potential antibacterial effects and spectrum of activity.

## Authors’ Contributions

We declare that this work was done by the authors named in this article and all liabilities pertaining to claims relating to the content of this article will be borne by them. F JB, S H, R S, and Y HY developed the idea to conduct this review. L JS, C YY, S WX, A VH, L YH, and T R performed the literature search and organized the findings under supervision of FJB, SH, RS, and YHY. The manuscript was written by L JS, C YY, S WX, A VH, L YH, and T R, and reviewed by F JB, S H, R S, and Y HY. L JS, C YY, F JB, S H, R S, and Y HY have critically revised the manuscript. All authors read and approved the final manuscript.

## Conflicts of Interest

The authors declare no conflict of interest.

## References

[1] Teoh L, Stewart K, Marino R, McCullough M (2018). Antibiotic resistance and relevance to general dental practice in Australia. Aust Dent J.

[2] Plackett B (2020). Why big pharma has abandoned antibiotics. Nature.

[3] C Reygaert W (2018). An overview of the antimicrobial resistance mechanisms of bacteria. AIMS Microbiol.

[4] Elshamy AA, Aboshanab KM (2020). A review on bacterial resistance to carbapenems: epidemiology, detection and treatment options. Futur Sci OA.

[5] Centre WM (2017). WHO publishes list of bacteria for which new antibiotics are urgently needed.

[6] Exner M, Bhattacharya S, Christiansen B, Gebel J, Goroncy-Bermes P, Hartemann P (2017). Antibiotic resistance: What is so special about multidrug-resistant Gram-negative bacteria?. GMS Hyg Infect Control.

[7] Ongenae V, Briegel A, Claessen D (2021). Cell wall deficiency as an escape mechanism from phage infection. Open Biol.

[8] Santajit S, Indrawattana N (2016). Mechanisms of antimicrobial resistance in ESKAPE pathogens. Biomed Res Int.

[9] Bush K, Jacoby GA (2010). Updated functional classification of beta-lactamases. Antimicrob Agents Chemother.

[10] Breijyeh Z, Jubeh B, Karaman R (2020). Resistance of Gram-negative bacteria to current antibacterial agents and approaches to resolve it. Molecules.

[11] Lee CR, Lee JH, Park M, Park KS, Bae IK, Kim YB (2017). Biology of Acinetobacter baumannii: Pathogenesis, antibiotic resistance mechanisms, and prospective treatment options. Front Cell Infect Microbiol.

[12] Pang Z, Raudonis R, Glick BR, Lin TJ, Cheng Z (2019). Antibiotic resistance in Pseudomonas aeruginosa: Mechanisms and alternative therapeutic strategies. Biotechnol Adv.

[13] Hsu LY, Apisarnthanarak A, Khan E, Suwantarat N, Ghafur A, Tambyah PA (2017). Carbapenem-resistant Acinetobacter baumannii and Enterobacteriaceae in south and southeast asia. Clin Microbiol Rev.

[14] Naas T, Dortet L I, Iorga B (2016). Structural and functional aspects of class a carbapenemases. Curr Drug Targets.

[15] Blanco P, Hernando-Amado S, Reales-Calderon JA, Corona F, Lira F, Alcalde-Rico M (2016). Bacterial multidrug efflux pumps: Much more than antibiotic resistance determinants. Microorganisms.

[16] Li-Jing Zhu, Yan Pan, Chun-Yan Gao P-FH (2020). Distribution of carbapenemases and efflux pump in carbapenem-resistance Acinetobacter baumannii-pubmed. Ann Clin Lab Sci.

[17] Wong MH yin, Chan BK wai, Chan EW chi, Chen S (2019). Over-expression of ISAba1-linked intrinsic and exogenously acquired OXA type carbapenem-hydrolyzing-class D-ß-lactamase-encoding genes is key mechanism underlying carbapenem resistance in Acinetobacter baumannii. Front Microbiol.

[18] Yoon EJ, Jeong SH (2021). Mobile carbapenemase genes in Pseudomonas aeruginosa. Front Microbiol.

[19] Meletis G, Exindari M, Vavatsi N, Sofianou D, Diza E (2012). Mechanisms responsible for the emergence of carbapenem resistance in Pseudomonas aeruginosa. Hippokratia.

[20] Kang AD, Smith KP, Eliopoulos GM, Berg AH, McCoy C, Kirby JE (2017). In vitro apramycin activity against multidrug-resistant Acinetobacter baumannii and Pseudomonas aeruginosa. Diagn Microbiol Infect Dis.

[21] Becker K, Aranzana-Climent V, Cao S, Nilsson A, Shariatgorji R, Haldimann K (2021). Efficacy of EBL-1003 (apramycin) against Acinetobacter baumannii lung infections in mice. Clin Microbiol Infect.

[22] Seifert H, Stefanik D, Sutcliffe JA, Higgins PG (2018). In vitro activity of the novel fluorocycline eravacycline against carbapenem non-susceptible Acinetobacter baumannii. Int J Antimicrob Agents.

[23] Solomkin J, Evans D, Slepavicius A, Lee P, Marsh A, Tsai L (2017). Assessing the efficacy and safety of eravacycline vs ertapenem in complicated intra-abdominal infections in the investigating Gram-negative infections treated with eravacycline (ignite 1) trial: A randomized clinical trial. JAMA Surg.

[24] Solomkin JS, Gardovskis J, Lawrence K, Montravers P, Sway A, Evans D (2019). IGNITE4: Results of a phase 3, randomized, multicenter, prospective trial of eravacycline vs meropenem in the treatment of complicated intraabdominal infections. Clin Infect Dis.

[25] Nguyen LP, Park CS, Pinto NA, Lee H, Seo HS, Vu TN (2021). In vitro activity of a novel siderophore-cephalosporin LCB10-0200 (GT-1), and LCB10-0200/avibactam, against carbapenem-resistant Escherichia coli, Llebsiella pneumoniae, Acinetobacter baumannii, and pseudomonas aeruginosa strains at a tertiary hospital in Korea. Pharmaceuticals.

[26] Nguyen M, Joshi SG (2021). Carbapenem resistance in Acinetobacter baumannii, and their importance in hospital-acquired infections: A scientific review. J Appl Microbiol.

[27] Oh SH, Park HS, Kim HS, Yun JY, Oh K, Cho YL (2017). Antimicrobial activities of LCB10-0200, a novel siderophore cephalosporin, against the clinical isolates of Pseudomonas aeruginosa and other pathogens. Int J Antimicrob Agents.

[28] Mmatli M, Mbelle NM, Maningi NE, Sekyere JO (2020). Emerging transcriptional and genomic mechanisms mediating carbapenem and polymyxin resistance in Enterobacteriaceae: A systematic review of current reports. mSystems.

[29] Kim S, Jin JS, Choi YJ, Kim J (2020). LysSAP26, a new recombinant phage endolysin with a broad spectrum antibacterial activity. Viruses.

[30] Breidenstein E (2019). Novel small-molecule inhibitors of bacterial lipoprotein transport against Enterobacteriaceae.

[31] Zhang Y, Zhao C, Wang Q, Wang X, Chen H, Li H (2020). Evaluation of the in vitro activity of new polymyxin B analogue SPR206 against clinical MDR, colistin-resistant and tigecycline-resistant Gram-negative bacilli. J Antimicrob Chemother.

[32] Brown P, Abbott E, Abdulle O, Boakes S, Coleman S, Divall N (2019). Design of next generation polymyxins with lower toxicity: The discovery of SPR206. ACS Infect Dis.

[33] Grosser L, Heang K, Teague J, Warn P, Corbett D, Dawson MJ RA (2018). In vivo efficacy of SPR206 in murine lung and thigh infection models caused by multidrug resistant pathogens Pseudomonas aeruginosa and Acinetobacter baumannii. ECCMID.

[34] Phase 1 Study of PK and Safety of SPR206 in Subjects With Various Degrees Of Renal Function.

[35] Study to Assess the Intrapulmonary Pharmacokinetics of SPR206 in Healthy Volunteers.

[36] Sobke A, Makarewicz O, Baier M, Bär C, Pfister W, Gatermann SG (2018). Empirical treatment of lower urinary tract infections in the face of spreading multidrug resistance: In vitro study on the effectiveness of nitroxoline. Int J Antimicrob Agents.

[37] Fuchs F, Becerra-Aparicio F, Xanthopoulou K, Seifert H, Higgins PG (2022). In vitro activity of nitroxoline against carbapenem-resistant Acinetobacter baumannii isolated from the urinary tract. J Antimicrob Chemother.

[38] Hackel MA, Tsuji M, Yamano Y, Echols R, Karlowsky JA, Sahm DF (2018). In vitro activity of the siderophore cephalosporin, cefiderocol, against carbapenem-nonsusceptible and multidrug-resistant isolates of Gram-negative bacilli collected worldwide in 2014 to 2016. Antimicrob Agents Chemother.

[39] Available from: URL: https://clinicaltrials.gov/ct2/show/NCT02321800 A Study of Efficacy and Safety of Intravenous Cefiderocol (S-649266) Versus Imipenem/Cilastatin in Complicated Urinary Tract Infections - Full Text View - ClinicalTrials.

[40] Study of Cefiderocol (S-649266) or Best Available Therapy for the Treatment of Severe Infections Caused by Carbapenem-resistant Gram-negative Pathogens.

[41] Plazomicin (2022). National Center for Biotechnology Information.

[42] Connolly LE, Riddle V, Cebrik D, Armstrong ES, Miller LG (2018). A multicenter, randomized, double-blind, phase 2 study of the efficacy and safety of plazomicin compared with levofloxacin in the treatment of complicated urinary tract infection and acute pyelonephritis. Antimicrob Agents Chemother.

[43] NCT01096849 Study of plazomicin (ACHN-490) compared with levofloxacin for the treatment of complicated urinary tract infection and acute pyelonephritis.

[44] Achaogen I (2016). A Study of Plazomicin Compared With Colistin in Patients With Infection Due to Carbapenem-Resistant Enterobacteriaceae (CRE)(CARE).

[45] Therapeutics S (2020). A First in Human Study of the Safety and Tolerability of Single and Multiple Doses of SPR206 in Healthy Volunteers. clinicaltrials.gov.

[46] Zhou X, Willems RJL, Friedrich AW, Rossen JWA, Bathoorn E (2020). Enterococcus faecium: From microbiological insights to practical recommendations for infection control and diagnostics. Antimicrob Resist Infect Control.

[47] Rivera AM, Boucher HW (2011). Current concepts in antimicrobial therapy against select gram-positive organisms: Methicillin-resistant Staphylococcus aureus, penicillin-resistant pneumococci, and vancomycin-resistant enterococci. Mayo Clin Proc.

[48] O’Driscoll T, Crank CW (2015). Vancomycin-resistant enterococcal infections: epidemiology, clinical manifestations, and optimal management. Infect Drug Resist.

[49] Zerrouki H, Rebiahi SA, Hadjadj L, Ahlem F, Elhabiri Y, Sedrati T (2021). High frequency and diversity of Vancomycin-resistant Enterococci (VRE) in algerian healthcare settings. Infect Genet Evol.

[50] Hollenbeck BL, Rice LB (2012). Intrinsic and acquired resistance mechanisms in enterococcus. Virulence.

[51] Smith TT, Tamma PD, Do TB, Dzintars KE, Zhao Y, Cosgrove SE (2018). Prolonged linezolid use is associated with the development of linezolid-resistant Enterococcus faecium. Diagn Microbiol Infect Dis.

[52] Klare I, Fleige C, Geringer U, Thürmer A, Bender J, Mutters NT (2015). Increased frequency of linezolid resistance among clinical Enterococcus faecium isolates from German hospital patients. J Glob Antimicrob Resist.

[53] Chen H, Wu W, Ni M, Liu Y, Zhang J, Xia F (2013). Linezolid-resistant clinical isolates of enterococci and Staphylococcus cohnii from a multicentre study in China: Molecular epidemiology and resistance mechanisms. Int J Antimicrob Agents.

[54] Olearo F, Both A, Belmar Campos C, Hilgarth H, Klupp EM, Hansen JL (2021). Emergence of linezolid-resistance in vancomycin-resistant Enterococcus faecium ST117 associated with increased linezolid-consumption. Int J Med Microbiol.

[55] Ma Z, Lynch AS (2016). Development of a dual-acting antibacterial agent (TNP-2092) for the treatment of persistent bacterial infections. J Med Chem.

[56] Yuan Y, Wang X, Xu X, Liu Y, Li C, Yang M (2020). Evaluation of a dual-acting antibacterial agent, TNP-2092, on gut microbiota and potential application in the treatment of gastrointestinal and liver disorders. ACS Infect Dis.

[57] TNP-2092 to Treat Acute Bacterial Skin and Skin Structure Infection.

[58] Kaminishi T, Schedlbauer A, Ochoa-Lizarralde B, Astigarraga E de, Çapuni R, Yang F (2018). The third-generation tetracycline KBP-7072 exploits and reveals a new potential of the primary tetracycline binding pocket. bioRxiv.

[59] Huband MD, Mendes RE, Pfaller MA, Lindley JM, Strand GJ, Benn VJ (2020). In vitro activity of KBP-7072, a novel third-generation tetracycline, against 531 recent geographically diverse and molecularly characterized Acinetobacter baumannii species complex isolates. Antimicrob Agents Chemother.

[60] Safety, Tolerability and Pharmacokinetics of KBP-7072.

[61] A Multiple Ascending Dose Study to Investigate Safety of KBP-7072 in Healthy Subjects.

[62] Zhang B, Wang Y, Chen Y, Yang F (2016). Single ascending dose safety, tolerability, and pharmacokinetics of KBP-7072, a novel third generation tetracycline. Open Forum Infect Dis.

[63] Online D (2021). CRS-3123. DrugBank Online.

[64] Lomeli BK, Galbraith H, Schettler J, Saviolakis GA, El-Amin W, Osborn B (2019). Multiple-ascending-dose phase 1 clinical study of the safety, tolerability, and pharmacokinetics of CRS3123, a narrow-spectrum agent with minimal disruption of normal gut microbiota. Antimicrob Agents Chemother.

[65] Critchley IA, Green LS, Young CL, Bullard JM, Evans RJ, Price M (2009). Spectrum of activity and mode of action of REP3123, a new antibiotic to treat Clostridium difficile infections. J Antimicrob Chemother.

[66] Ochsner UA, Bell SJ, O’Leary AL, Hoang T, Stone KC, Young CL (2009). Inhibitory effect of REP3123 on toxin and spore formation in Clostridium difficile, and in vivo efficacy in a hamster gastrointestinal infection model. J Antimicrob Chemother.

[67] (2017). National Institute of Allergy and Infectious Diseases (NIAID). Phase I Trial of a Single Dose of CRS3123.

[68] Nayak SU, Griffiss JML, Blumer J, O’Riordan MA, Gray W, McKenzie R (2017). Safety, tolerability, systemic exposure, and metabolism of CRS3123, a methionyl-tRNA synthetase inhibitor developed for treatment of clostridium difficile, in a phase 1 study. Antimicrob Agents Chemother.

[69] A Multiple Ascending Dose Study of KBP-7072 in Healthy Subjects - Full Text View - ClinicalTrials.

[70] Guo Y, Song G, Sun M, Wang J, Wang Y (2020). Prevalence and therapies of antibiotic-resistance in Staphylococcus aureus. Front Cell Infect Microbiol.

[71] Keseru JS, Gál Z, Barabás G, Benko I, Szabó I (2005). Investigation of beta-Lactamases in clinical isolates of Staphylococcus aureus for further explanation of borderline methicillin resistance. Chemotherapy.

[72] Foster TJ (2019). Can β-lactam antibiotics be resurrected to combat MRSA?. Trends Microbiol.

[73] Gao Y, Chen Y, Cao Y, Mo A, Peng Q (2021). Potentials of nanotechnology in treatment of methicillin-resistant Staphylococcus aureus. Eur J Med Chem.

[74] Cong Y, Yang S, Rao X (2019). Vancomycin resistant Staphylococcus aureus infections: A review of case updating and clinical features. J Adv Res.

[75] Conly JM, Johnston BL (2002). VISA, hetero-VISA and VRSA: The end of the vancomycin era?. Can J Infect Dis.

[76] Sieradzki K, Tomasz A (1999). Gradual alterations in cell wall structure and metabolism in vancomycin-resistant mutants of Staphylococcus aureus. J Bacteriol.

[77] Liu WT, Chen EZ, Yang L, Peng C, Wang Q, Xu Z (2021). Emerging resistance mechanisms for 4 types of common anti-MRSA antibiotics in Staphylococcus aureus: A comprehensive review. Microb Pathog.

[78] Sohlenkamp C, Geiger O (2016). Bacterial membrane lipids: Diversity in structures and pathways. FEMS Microbiol Rev.

[79] Ernst CM, Staubitz P, Mishra NN, Yang SJ, Hornig G, Kalbacher H (2009). The bacterial defensin resistance protein MprF consists of separable domains for lipid lysinylation and antimicrobial peptide repulsion. PLOS Pathog.

[80] Hawser S, Gueny M, Le Bras Ch, Morrissey I, Valmont Th, Magnet FW S (2016). Activity of debio 1452 against Staphylococcus spp collected in 2013/2014. Debiopharm Group.

[81] Menetrey A, Janin A, Pullman J, Scott Overcash J, Haouala A, Leylavergne F (2019). Bone and joint tissue penetration of the staphylococcus-selective antibiotic afabicin in patients undergoing elective hip replacement surgery. Antimicrob Agents Chemother.

[82] Drug Penetration Into Bone After Repeated Oral Administration of Debio 1450 to Patients Undergoing Hip Replacement Surgery - Full Text View - ClinicalTrials.

[83] Study of Debio 1450 for Bacterial Skin Infections - Full Text View - ClinicalTrials.

[84] Study to Assess Safety, Tolerability and Efficacy of Afabicin in The Treatment of Participants With Bone or Joint Infection Due to Staphylococcus - Full Text View - ClinicalTrials.

[85] Biedenbach DJ, Bouchillon SK, Hackel M, Miller LA, Scangarella-Oman NE, Jakielaszek C (2016). In vitro activity of gepotidacin, a novel triazaacenaphthylene bacterial topoisomerase inhibitor, against a broad spectrum of bacterial pathogens. Antimicrob Agents Chemother.

[86] A Study to Evaluate Efficacy and Safety of Gepotidacin in the Treatment of Uncomplicated Urinary Tract Infection (UTI) - Full Text View - ClinicalTrials.

[87] A Study Evaluating Efficacy and Safety of Gepotidacin Compared With Ceftriaxone Plus Azithromycin in the Treatment of Uncomplicated Urogenital Gonorrhea - Full Text View - ClinicalTrials.

[88] Saravolatz LD, Pawlak J (2019). Delafloxacin activity against Staphylococcus aureus with reduced susceptibility or resistance to methicillin, vancomycin, daptomycin, or linezolid. Open Forum Infect Dis.

[89] Delafloxacin Versus Vancomycin and Aztreonam for the Treatment of Acute Bacterial Skin and Skin Structure Infections - Full Text View - ClinicalTrials.

[90] Dalbavancin For The Treatment of Gram Positive Osteoarticular Infections - Full Text View - ClinicalTrials.

[91] Turner NA, Zaharoff S, King H, Evans S, Hamasaki T, Lodise T (2022). Dalbavancin as an option for treatment of S aureus bacteremia (DOTS): Study protocol for a phase 2b, multicenter, randomized, open-label clinical trial. Trials.

